# What research agenda could be generated from the European General Practice Research Network concept of Multimorbidity in Family Practice?

**DOI:** 10.1186/s12875-015-0337-3

**Published:** 2015-09-17

**Authors:** JY Le Reste, P. Nabbe, H. Lingner, D. Kasuba Lazic, R. Assenova, M. Munoz, A. Sowinska, C. Lygidakis, C. Doerr, S. Czachowski, S. Argyriadou, J. Valderas, B. Le Floch, J. Deriennic, T. Jan, E. Melot, P. Barraine, M. Odorico, C. Lietard, P. Van Royen, H. Van Marwijk

**Affiliations:** ERCR SPURBO, Department of General Practice, Université de Bretagne Occidentale, Brest, France; Centre for Public Health and Healthcare, Hannover Medical School, Hannover, Germany; Department of General Practice, University of Zagreb, Zagreb, Croatia; Department of General Practice, University of Plovdiv, Plovdiv, Bulgaria; IDIAP Jordi GOL Unitat de Support a la Recerca, Barcelona, Spain; Department of English, Nicolaus Copernicus University, Torun, Poland; Associazione Italiana Medici di Famiglia (AIMEF), Bologna, Italy; Allgemein Medizin Hochschule Göttingen, Göttingen, Germany; Department of Family Doctor, University Nicolaus Copernicus, Torun, Poland; The Greek Association of General Practitioners (ELEGEIA), Thessaloniki, Greece; Patient Centred Care Lead, University of Exeter Collaboration for Academic Primary Care (APEx), University of Exeter Medical School, Exeter, UK; Department of Public Health, Université de Bretagne Occidentale, Brest, France; Department of Primary and Interdisciplinary Care, Faculty of Medicine and Health Sciences, Universiteit Antwerpen, Antwerpen, Belgium; Primary Care Research Center, Williamson Building, Oxford Road, Manchester, UK

## Abstract

**Background:**

Multimorbidity is an intuitively appealing, yet challenging, concept for Family Medicine (FM). An EGPRN working group has published a comprehensive definition of the concept based on a systematic review of the literature which is closely linked to patient complexity and to the biopsychosocial model. This concept was identified by European Family Physicians (FPs) throughout Europe using 13 qualitative surveys. To further our understanding of the issues around multimorbidity, we needed to do innovative research to clarify this concept. The research question for this survey was: what research agenda could be generated for Family Medicine from the EGPRN concept of Multimorbidity?

**Methods:**

Nominal group design with a purposive panel of experts in the field of multimorbidity. The nominal group worked through four phases: ideas generation phase, ideas recording phase, evaluation and analysis phase and a prioritization phase.

**Results:**

Fifteen international experts participated. A research agenda was established, featuring 6 topics and 11 themes with their corresponding study designs. The highest priorities were given to the following topics: measuring multimorbidity and the impact of multimorbidity. In addition the experts stressed that the concept should be simplified. This would be best achieved by working in reverse: starting with the outcomes and working back to find the useful variables within the concept.

**Conclusion:**

The highest priority for future research on multimorbidity should be given to measuring multimorbidity and to simplifying the EGPRN model, using a pragmatic approach to determine the useful variables within the concept from its outcomes.

## Background

The number of people suffering from multiple conditions (multimorbidity) is rising rapidly especially in family medicine (FM) [1]. The concept of multimorbidity was first described in the 1970s [2]. It was, at that time, an addition to the concept of comorbidity, with the intention of looking at all the conditions in one individual [3–5]. Nevertheless, the concept remained unclear, especially for research and practical purposes [6, 7]. The World Health Organisation (WHO), in 2008, tried to clarify the concept and defined Multimorbidity as people being affected by two or more chronic health conditions [8]. The intention of the WHO was to look at all conditions in one individual that could impact on that individual’s global health status. However the word’condition’ was not sufficiently clear for research or practical purposes (for instance, whether a treated disease was a ‘condition’ in this sense), and could lead to numerous interpretations.

Despite those interpretations, multimorbidity is a very interesting and challenging concept, particularly for FM and long-term care, given the increasing prevalence of chronic illness in an aging population across all countries. It is closely related to a global or comprehensive view of the patient, which is a core competency of FM, as defined for instance by the World Organization of National Colleges, Academies and Academic Associations of General Practitioners/Family Physicians (WONCA) [9]. It is also a global ‘functional’ or ‘goal-oriented’ view (useful for Long-Term Care) versus a ‘disease’ centered point of view (useful for acute care). Nevertheless, the disease centered point of view remains the basis of most clinical guidelines even if it is not fully applicable in FM [10, 11].

The European General Practice Research Network (EGPRN) was very interested in the concept of multimorbidity as this network is committed to concepts that could advance research and practice in primary care throughout Europe. The EGPRN has created a research agenda specifically designed for methodological and instrumental research, which includes the development of primary care epidemiology, focusing on patient-centered health [12]. A clear definition of the concept of multimorbidity (i.e., one which is both understandable and usable for further collaborative research) is an important objective for a research network of this type. It aims to help researchers in FM to investigate the complexity of patients’ conditions and their overall impact on patients’ health. A clear definition of multimorbidity could be an additional tool for Family Physicians (FPs), enabling them to identify frail patients and prevent decompensation [13]. A specific research agenda could be developed for multimorbidity.

A research group, including 9 national groups, all active within the EGPRN, has created a research community for the purpose of clarifying the concept of multimorbidity for FM throughout Europe [14]. An initial review, presented in an EGPRN meeting in spring 2011 [15], identified more than one hundred different definitions used by academic researchers. Such a large number of definitions added more confusion than clarification to the discussion and led the group to the production of a comprehensive definition of the concept of multimorbidity with the help of a systematic review of literature [16].

It was then necessary to assess whether Family Physicians (FPs) recognized this concept, which had emerged from medical research, as applicable to their complex patients. FPs are well-placed to identify this concept as they aim to be more aware of their patients’ expectations than other specialists [17] and they are used to dealing with complex patients [18, 19]. In order to make this assessment, the comprehensive definition of Multimorbidity was carefully translated into 10 European languages with the help of a forward-backward translation procedure using a Delphi consensus methodology [20]. Those translated definitions were presented to European FPs with 13 consecutive qualitative surveys, designed to check how the FPs experienced and worked with the developed concept of multimorbidity and whether this was fully consistent with the definition. European FPs clarified the concept and added the role of gut feelings, core competencies of FM, and patient and doctor experience, to the management of Multimorbidity. A comprehensive concept of multimorbidity for FM was then established.

Researchers in the field of multimorbidity explored innovative research topics, themes, questions and appropriate design formats in relation to the concept of Multimorbidity for FM, leading to the question: what research agenda could be generated for Family Medicine from the EGPRN concept of Multimorbidity?

## Methods

For this study we used a qualitative research method, i.e., a nominal group technique (NGT) [21]. This technique was chosen because multimorbidity is conceptually complex. The NGT enables researchers to gather information from relevant experts [22, 23]. NGT facilitates creative problem solving by means of judgmental decision making in situations where routine answers are inadequate [24]. With an NGT, it is possible to plan research through group meetings or by email [25]. The ethical committee of the “université de Bretagne occidentale” gave the ethical approval for the whole process as this university led the study. Informed consent was obtained from all participants even if it was a non-interventional study. NGT is a well-known technique that has already been used by members of the research group [26].

With an international group, adaptations were needed and an email system was used as it had already been successfully employed in several earlier studies [27–29]. An NGT involves four phases: *ideas generation phase, ideas recording phase, evaluation and analysis phase* and a *prioritization phase*.

### Type of participants and selection of experts

An international panel of experts in the field of multimorbidity was purposively sampled. The NGT does not require a fixed number of experts in order to be valid but does require relevant experts. To be relevant, those experts should have prior experience in the field of the research in question (multimorbidity, FM, patient-centered care). The group was selected from three backgrounds: The EGPRN, the Threads and Yarns network members (a group designed for research into the field of multimorbidity), and researchers in multimorbidity from Polish, Dutch and British Universities. The group was made up of FM clinicians, researchers in FM and linguistics, methodologists and epidemiologists. Some individuals were involved in two different groups. 18 experts were approached by email. 18 were willing to cooperate and received written information. We invited them to participate and 15 accepted. Reasons for declining were prior engagements and illness.

### NGT sessions

For *ideas generation,* experts were asked to read the publications about the concept of Multimorbidity produced by the EGPRN (protocol [14], systematic review [16], translations [20] and qualitative surveys). Then they were asked to take time and write down what they regarded as the main research questions relating to the “concept of Multimorbidity issued by the EGPRN”. At the same time, they were asked to present an appropriate design for each research question, with a commentary to make it easily understandable by each participant. Each expert could produce a maximum of five propositions, arising from that definition, with a design and a commentary for each. The aim was to leave this process wide open in order to elicit the widest possible variety of responses.

In the *ideas recording* phase participants were engaged in a round-robin feedback system by email. All the propositions were sorted, with their designs and commentaries, and classified to detect duplicates. Duplicates were summarized and sent back to the planners to check that they were compatible with the planners’ initial intentions. Where necessary, corrections were made to the questions, designs and commentaries.

The *evaluation and analysis* phase was undertaken to identify and clarify research topics and themes and to develop designs. All the propositions were classified into research topics and themes and then summarized by an independent group of 6 researchers from the SPURBO research team (Université de Bretagne Occidentale). These 6 researchers’ propositions were sent to all participants for agreement before any further development. Then designs were developed, for each research theme and topic, by their proposers. The designs were sent back to all the participants for analysis. Where necessary, it was possible to have open discussions, by email, between members so that submitted propositions could be clarified. These emails were sent out to all the participants so that all the members received clarification on each proposition.

The intention of the *prioritization* phase was to aggregate the judgment of the experts in order to determine the relative importance of the research questions, and corresponding designs for future research relating to the concept of Multimorbidity produced by the EGPRN. All participants were asked to rank the propositions, according to the level of importance they attached to them, for FM and patient centered care. They had the opportunity to apply 3 scores: a score of 5 points for one proposition, a score of 3 points for another and a score of 1 point for the final proposition. A prioritized list of all the propositions was drawn up from these scores. This prioritized list was sent back to all the participants in order to evaluate the procedure and the outcomes and to collect objections, should any arise.

## Results

### Description of participants

In total, 15 experts participated in the study, 7 men and 8 women from different countries, including Bulgaria, Croatia, France, Germany, Greece, Italy, Poland, Spain and the UK. Their average age was 49 years (ranging from 35 to 62 years). All clinicians (14 out of 15) had an average of 19.8 years’ practice experience (ranging from 4 to 33 years), with 10 working in group practices, mostly in urban areas. Methodologists, linguists and epidemiologists were also experienced, with an average of 38.5 published articles. They all had experience of publishing articles, with an average of 34 published articles (ranging from 6 to70), of which an average of 16 were in English (ranging from 3 to 65). Participants had had an average of 5 articles published with multimorbidity as a major topic (ranging from 1 to15).

### Description of results

The ideas generation and ideas recording phase produced 61 research questions and study designs after duplicates had been withdrawn. Although there was considerable overlap in the ideas, the evaluation and analysis phase aggregated those 61 research questions into 11 themes, including 6 major topics, with their attached research questions and designs. The prioritization phase produced a consensual ranking of the topics and themes. The total score possible, by adding up the points of all 15 experts, was 135. Topics, themes and study design, with their ranking, are described in Table 1 below.

**Table 1:** Topics, Themes and design ranked by participants’ votes

Topics, themes and designs for multimorbidity Agenda				
Topics	Themes	Study design formats	Points per theme	Points per topic
Measuring multimorbidity	Developing or finding measurement tool of multimorbidity	Expert consensus and cross sectional	31	49
	Epidemiology of Multimorbidity	Expert consensus or cross sectional or cohort	18	
Impact of (or on) Multimorbidity	Impact of multimorbidity on frailty or health outcomes, or cost of care, or health service utilization, or depression.	Cohort	38	40
	Impact of socioeconomic status on multimorbidity	Cross sectional or cohort	2	
Management of multimorbidity	Multimorbidity management in General Practice	Expert consensus or cross sectional	16	24
	Patient Doctor relationship evaluation in the management of multimorbidity	Cross sectional	5	
	FP workload and burden of multimorbidity	Cross sectional	3	
Patient perspective	Multimorbid patient's perspective	Qualitative study (semi directed interviews or focus groups)	8	13
	Burden of diseases effect on multimorbidity from patient’s perspective	Qualitative study and expert consensus	5	
Links between complexity and multimorbidity	Multimorbidity definition as a help to detect complexity	Cross sectional	6	6
Stakeholders’ perspective	Consensus for multimorbidity research according to stakeholders’ interest	Expert consensus	3	3
Total votes			135	135

Experts had 9 points to allocate (5 for their first top theme, 3 for the second, 1 for the third). They were 15 experts with a maximum of 135 points to allocate. Top three research themes are the first three. All the others are secondary research themes

The research questions suggested for the multimorbidity research agenda are listed, by topic, in Table 2 below.

**Table 2:** Proposed research questions for the different topics and themes

Proposed research questions for the different topics and themes		
Topics	Themes	Research question examples
Measuring multimorbidity	Developing or finding measurement tool for multimorbidity	What tool could be designed using the definition of multimorbidity to enhance medical decision making (including shared decision-making)? Then design an application to be implemented in electronic medical records and evaluate that tool’s effectiveness in decision-making.
	Epidemiology of multimorbidity	Are there clear trends in the development of multimorbidity at the individual (patient) level? What are the predictors of multimorbidity and are there any specific patterns of accumulation? Are there specific patterns and conditions which are likely to accelerate the development of multimorbidity? Evaluation of MM in EU countries? Would it be possible to measure the different levels of multimorbidity in order to describe the patient's complexity?
Impact of multimorbidity	Impact of multimorbidity on patient or health service outcomes.	Have the different criteria included in the definition of multimorbidity different predictive powers in terms of patient outcomes (Mortality, health status, frailty, health outcomes decline, poly-pharmacy, depression) or health system outcomes (cost of care or poly-pharmacy or health services utilization)? With an additional question for depression: is depression a specific factor or is it related to Pain in Multimorbidity measurement?
	Impact of socioeconomic status on multimorbidity	What is the Impact of multimorbidity on particular groups (low socioeconomic status, addictive persons, societies in economic crisis)? What is the role of socioeconomic differences in multimorbidity?
Management of multimorbidity	Multimorbidity management in General Practice	What are the methods to promote medical audit in patients with multimorbidity? How does multimorbidity influence FP management? How can medical records of co-morbid patients be improved?
	Patient Doctor relationship evaluation in the management of multimorbidity	Is the doctor- patient relationship a modifying factor in the concept of multimorbidity? This study will take into account communicative challenges, including, not only FPs’ communicative skills/ communicative competence, but in particular, FPs’ ability to cope with their own emotions and the patients’ emotions.
	FP workload and burden of multimorbidity	Do FPs of multimorbid patients have an unchanged quality of life when their patients' multimorbidity increases? Selecting groups of FPs according to their patients’ multimorbidity, using the definition of multimorbidity and comparing their quality of life.
Patient perspective	Multimorbid patient's perspective	How do patients conceptualize the multimorbidity condition using their own language, concepts, metaphors, and expectations? With the intention of looking at the impact of multimorbidity on patients’ experiences of self-management and health care
	Burden of diseases effect on multimorbidity (from patient’s perspective) and prevention strategies	What is the role of the patient perceived burden of diseases into the multimorbidity definition? Is the burden of disease, as perceived by the patient, a modifier of multimorbidity or should it be included in the defining illnesses? Is it possible to assert that only diseases with a burden should be considered to contribute to multimorbidy? (For example, not a healed cataract or stabilized hypertension).
Links between complexity and multimorbidity	Multimorbidity definition as a help in detecting complexity	How could the concept of multimorbidity improve the ability to detect complex patients in family medicine? As risk factors, guidelines and recommendation for secondary prevention are well defined for cardiovascular diseases and diabetes mellitus, the aim of the study might be to identify the level of comorbidity (in terms of complexity) in these patients, to define barriers, to follow preventive activities which are well defined by European associations of cardiologists and diabetologists. It can be measured from the FP’s perspective, and specific diseases perspective and from the patient’s perspective), with the aim of formulating more specific recommendation for the preventive and curative care of multimorbid patients with cardiovascular diseases.
Stakeholders’ perspective	Consensus for multimorbidity research according to stakeholders’ interest	Are stakeholders interested in the patient’s perspective (multimorbidity: perceived severity and grading, self-management and individualized patient-centered plan) or health professional’s perspective (operational definition of MM, improvement of clinical decision support, practice-based guidelines in multimorbidity and poly-pharmacy) or in research perspective (gaps in multimorbidity research, consensus-based set of recommendations for inclusion and reporting of multimorbid patients in Randomized Controlled Trials, and for addressing multimorbidity in Clinical Practice Guidelines)

## Discussion

### Main findings

With the help of a nominal group technique, the research team was able to establish a research agenda on Multimorbidity in Family Practice. 6 topics and 11 themes were listed with their corresponding designs (see Table 1). Overall, the highest priorities were given to the following topics: measuring Multimorbidity and the impact of Multimorbidity (see Table 2).

The measurement of Multimorbidity is seen as the most important topic when totaling the votes for the different themes included. The impact of Multimorbidity on patient or health outcomes is seen by the research group as the most important theme for future research, even if it is included in the second topic to research (Impact of Multimorbidity). The management of multimorbidity in practice is seen as the third most important topic.

### Interpretation

The measurement of Multimorbidity has been explored considerably over the past 10 years and has often been divergent as the authors did not use the same definition of this concept [7, 30]. With the EGPRN concept of multimorbidity, this pitfall could be avoided, especially if an expert consensus could be obtained on the effect of the burden of diseases on multimorbidity (see Table 1). The effect of the burden of diseases on multimorbidity was a secondary research theme in this survey (7^th^ theme to be researched) but could also be of major importance for the measurement of Multimorbidity. Although it has already been the subject of many studies [30, 31], practitioner input has, so far, been limited. Most of our experts are also practicing FPs and confirm the importance of this topic.

An alternative way of measuring multimorbidity, for this study’s experts, was to determine the impact of multimorbidity on negative health outcomes (such as frailty, depression, cost of care, health service use). The impact of multimorbidity was, according to the experts in this study, for the patient, the clinical, negative result of being Multimorbid. It is a reverse way of determining multimorbidity. The burden of diseases could determine whether a patient is multimorbid and the impact of multimorbidity will determine the effect of multimorbidity on a patient’s life. This alternative route could lead a research team to define the most effective variables that contribute to the concept of multimorbidity to help prevent those negative outcomes. In addition, it could simplify the concept when it is aimed at a specific outcome, to help clinicians in everyday practice. This was an attractive issue for most experts. It could resolve the debate about measuring a concept as broad as multimorbidity seems to be. Many studies have been conducted to assess the relationship between multimorbidity and health outcomes but without solving the problem of the meaning and the intensity of that relationship [32, 33].

The results underline the usefulness of the patient’s perception of his/her own multimorbidity which could be an alternative way to weigh and measure multimorbidity. This perception has rarely been explored [34]. The experts emphasized this fourth topic for future research and its importance for them. They were stimulated by their own practice experience and by their need to integrate the patients’ perspective as a key element of the decision making process. They felt that the patient’s perception was one way to enhance the coping strategies of the patient which is of importance in the EGPRN definition of multimorbidity: to lower the level of multimorbidity.

How to improve the management of multimorbidity in everyday practice seemed of importance to our experts, as a research theme, and this management focus could be linked to patient complexity, as both are important issues for family medicine. Using the concept of multimorbidity as an aid for detecting and managing patient complexity may be a new pathway for research.

The final topic took into account all the stakeholders’ perspectives, which should be incorporated into all the research, as stakeholders reflect population needs [4].

### Strengths and limitations

Nominal group technique has already been used as a method to generate ideas for study designs [35]. It is an efficient technique to gather specific ideas about difficult research questions. The benefit of nominal group technique is that all experts have an equal opportunity to participate and influence the decision. It reduces the conforming common influence of face-to-face group meetings.

The study team tried to be as open as possible in the data collection. Information bias was limited as all the experts had open access to all the information. This bias was still possible because their prior researches in the field of multimorbidity could influence their prioritization. To handle that possible bias, throughout the entire process, the research team stimulated open discussion and interaction through regular face-to-face meetings. Selection bias was limited by the use of a pan-European panel of researchers, with real experience of multimorbidity research, assessed by specific publications, as well as experience in practicing as FPs.

### Implications

Results allow researchers to start relevant and, it is hoped, high-quality studies using the EGPRN concept of multimorbidity, avoiding difficulties previously encountered in research into multimorbidity, to detect and measure it in practice. They highlight the connection between multimorbidity and complexity which could lead to specific recommendations for the complex patients, often seen in Family Medicine, whose situation has rarely been explored in research up to now. They increase the possibility of using reverse methods, starting from the outcomes, or the patient’s perspective, or the stakeholder’s perspective, and working back to the variables in the concept which are useful for research, in order to create pragmatic models for multimorbidity.

They also open up a new aim for practitioners: multimorbidity could help them in managing patients, by using a more holistic and goal-oriented approach as the EGPRN concept of multimorbidity is closely related to the biopsychosocial model [36]. The aim would be to place the exchange with the patient at the center of the clinical consultation process rather than allocate it a supporting role [37].

They also open new perspectives in medical education, focusing on the patient’s perspective which is a core competency for Family Practice, as specified in the WONCA Family Practice competencies [9].

## Conclusion

Using a Nominal Group technique, a research agenda is now available for further research into Multimorbidity. The highest priority should be given to measuring multimorbidity and to simplifying this model, using the outcomes of a pragmatic approach to determine the useful variables of this concept.

## Abbreviations

FM: Family medicine
FPs: Family physicians
EGPRN: European General Practice Research Network
NGT: Nominal Group Technique
WONCA: World Organization of National Colleges, Academies and Academic Associations of General Practitioners/Family Physicians
